# EORTC 2238 “De-Escalate”: a pragmatic trial to revisit intermittent androgen deprivation therapy in the era of new androgen receptor pathway inhibitors

**DOI:** 10.3389/fonc.2024.1391825

**Published:** 2024-05-08

**Authors:** Guillaume Grisay, Fabio Turco, Saskia Litiere, Béatrice Fournier, Anna Patrikidou, Enrique Gallardo, Ray McDermott, Ahu Alanya, Silke Gillessen, Bertrand Tombal

**Affiliations:** ^1^ Department of Medical Oncology, Centres Hospitaliers Universitaires HELORA, La Louvière, Belgium; ^2^ Oncology Institute of Southern Switzerland, Ente Ospedaliero Cantonale, Bellinzona, Switzerland; ^3^ Statistics Department, European Organization for Research and Treatment of Cancer (EORTC), Brussels, Belgium; ^4^ Medical Department, European Organization for Research and Treatment of Cancer (EORTC), Brussels, Belgium; ^5^ Genito-Urinary Oncology Group and Early Drug Development (DITEP), Gustave Roussy, Villejuif, France; ^6^ Department of Oncology, Parc Taulí Hospital Universitari, Institut d’Investigació i Innovació Parc Taulí (I3PT-CERCA), Universitat Autònoma de Barcelona, Sabadell, Spain; ^7^ Department of Medical Oncology, St Vincents University Hospital and Cancer Trials, Dublin, Ireland; ^8^ Quality of Life Department, European Organization for Research and Treatment of Cancer (EORTC), Brussels, Belgium; ^9^ Faculty of Biomedical Sciences, Università della Svizzera Italiana, Lugano, Switzerland; ^10^ Division of Urology, Cliniques Universitaires Saint Luc, Brussels, Belgium

**Keywords:** mHNPC, de-escalation, maximal androgen blockade, AR pathway inhibitor, quality of life

## Abstract

The landscape of treating metastatic prostate cancer has evolved with the addition of Androgen Receptor pathway inhibitor (ARPI) to Androgen Deprivation Therapy (ADT), significantly improving survival rates. However, prolonged use of these therapies introduces notable side effects, prompting a need to revisit intermittent treatment duration. The EORTC 2238 De-Escalate trial is a pragmatic trial seeking to reassess the role of intermittent therapy in patients undergoing maximal androgen blockade (MAB) for metastatic hormone naïve prostate cancer (mHNPC), i.e., the combination of ADT with an ARPI, with the aims of reducing side effects, enhancing Quality of Life (QoL) and optimizing resource usage, while maintaining oncological benefits.

## 1 Introduction

Androgen deprivation therapy (ADT) through surgical castration or GnRH analogs has been the standard of care treatment for advanced prostate cancer (PCa) for six decades. Seven trials have now demonstrated that combining ADT with one of the androgen receptor pathway inhibitors (ARPI) - abiraterone, apalutamide, enzalutamide, and darolutamide - significantly prolongs progression-free (PFS) and overall survival (OS) (1–7). Called maximal androgen blockade (MAB), the combination of ADT and an ARPI also delays the progression to a further line of treatment and deterioration of quality of life (QoL) by delaying symptomatic progression. Consequently, MAB has become the new standard of care for men with metastatic hormone-naïve prostate cancer (mHNPC), regardless of the tumor burden (high vs. low volume/risk), timing (*de novo* vs. metachronous), or the co-administration of docetaxel and/or prostate radiotherapy.

There is, however, a price to pay. In landmark trials, the treatment was administered continuously until progression or unacceptable toxicity, sometimes up to several years. The patient is thus exposed for extended periods to the well-known toxicities of ADT and, furthermore, to the specific side effects of ARPI.

Interestingly, prior to the era of the ARPI trials, intermittent ADT (iADT) was often used to alleviate the side effects of androgen deprivation. Three prospective trials have tested an iADT regimen in patients with newly diagnosed mHNPC. None of these trials showed a clear difference in OS of iADT versus continuous dosing (8–10). A 10% difference in mortality appeared to favor the continuous arm, however, this did not reach statistical significance. Non-inferiority cannot therefore be proved or disproved, nor can the superiority of either strategy be ruled out.

**Figure 1:**
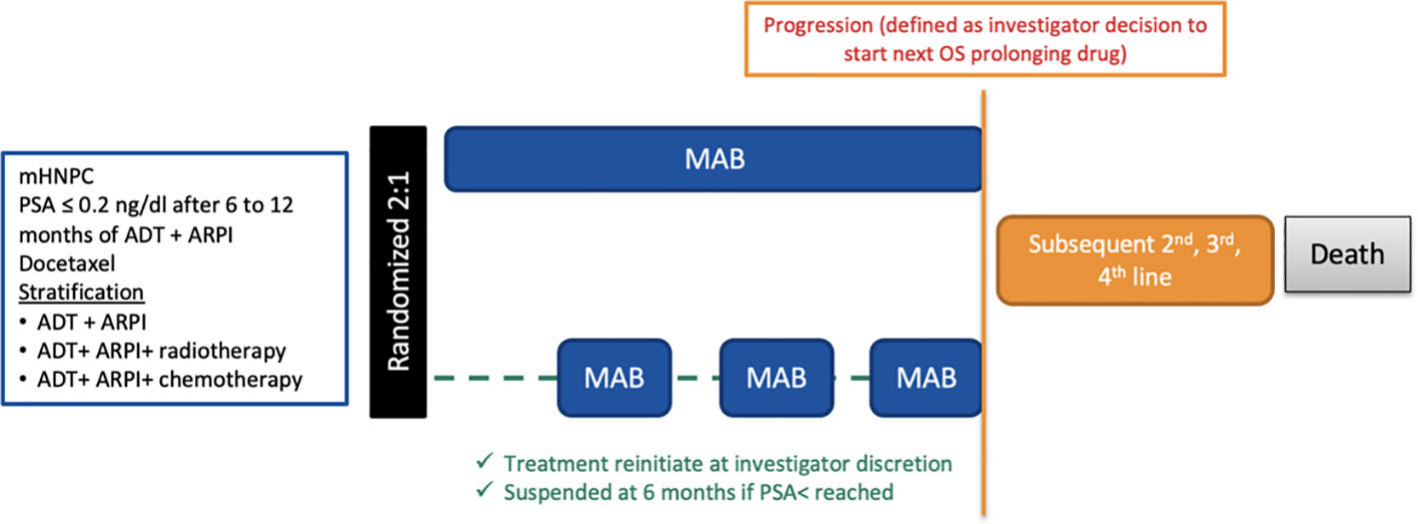
Trial scheme.

EORTC 2238 (De-Escalate) investigates the risk/benefit of intermittent treatment in patients on MAB for 6 to 12 months who have achieved a significant prostate-specific antigen (PSA) decline to ≤0.2 ng/ml (**Figure 1**). First demonstrated by Hussain, deep PSA response is a robust prognostic survival factor. Patients who are good responders are, therefore, likely to be exposed for longer periods to toxicities and side effects while deriving few benefits (11).

This trial is essential to reassess the benefit of intermittent treatment in patients receiving ADT and an ARPI as it could potentially reduce side-effects, improve QoL and reduce resource utilization.

## 2 The new treatment landscape, its benefits, and consequences

The development of four ARPI has profoundly reshaped the management of mHNPC. They are the CYP17A inhibitor (abiraterone acetate) and the AR antagonists (apalutamide, darolutamide and enzalutamide). Seven trials have demonstrated that combining any of these drugs to ADT ± docetaxel consistently reduces the risk of death by 20 to 40%, delays progression to the next line of treatment and improves health related QoL (HRQoL) by delaying symptomatic progression. Apalutamide (TITAN trial) and enzalutamide (ARCHES and ENZAMET) have been evaluated in patients with either high or low-volume disease (4–6). Darolutamide has been tested only with docetaxel (ARASENS) (12). Abiraterone acetate has been evaluated in high-risk disease in the LATITUDE trial, but has also been tested in broader disease settings in the STAMPEDE and PEACE-1 trials (1, 3).

Hence, MAB with one of these agents is the new standard of care for mHNPC, notwithstanding the volume (high vs. low volume/risk) and timing of metastasis (*de-novo* vs metachronous) of the disease (EAU guidelines).

During the most recent Advanced Prostate Cancer Consensus Conference (APCCC), 77% of panelists recommended radical local treatment of the primary tumor and ADT plus one of the ARPIs for treating synchronous, low-volume HNPC (13). Eighty-nine percent of panelists recommended ADT and an ARPI (with 40% of them adding docetaxel) to treat synchronous, high-volume HNPC.

The addition of an ARPI to continuous ADT increases OS therefore resulting in a much longer treatment duration, thus increasing the toxicity associated. A recent meta-analysis of studies conducted in non-metastatic castration-resistant PCa (nmCRPC) concluded that the addition of an ARPI to ADT is associated with a significantly increased risk of cardiovascular events (RR = 1.71; 95% CI 1.29-2.27) and grade 3-4 hypertension (RR = 1.53; 95% CI 1.19-1.97) (14). In another recent review, the use of an ARPI was associated with an increased risk of falls and fractures: all-grade falls (RR, 1.8; 95% CI, 1.42-2.24; p < 0.001); grade ≥ 3 fall (RR, 1.6; 95% CI, 1.27-2.08; p < 0.001); all-grade fractures (RR, 1.59; 95% CI, 1.35-1.89; p < 0.001); and likely grade ≥3 fracture (RR, 1.71; 95% CI, 1.12-2.63; p = 0.01) (15). Furthermore, the recent systematic review and meta-analysis by Nowakowska et al. showed an increased risk of cognitive toxic effects (risk ratio (RR) 2.10; 95%CI 1.30-3.38; p = 0.002) and fatigue (RR 1.34; 95%CI 1.16-1.54; p <.001) with the use of ARPIs (16).

## 3 The need to revisit Intermittent androgen deprivation therapy

Intermittent ADT (iADT) consists of interrupting ADT in patients with a significant PSA response after 6 to 12 months of treatment. This allows the testosterone to return to normal, side effects to resolve, and HRQoL to improve. Regular measurements of testosterone and PSA are performed. ADT is restarted, usually upon PSA rise to a value at the investigator’s discretion, with each trial using its own individual criteria. Multiple cycles of on- and off-treatment can be performed in this way.

Nine randomized trials have assessed the feasibility of iADT as a strategy to preserve efficacy while decreasing side effects (17). All these trials used a conditional randomization scheme as the patients had to have achieved a predefined decrease in PSA levels from baseline. Three trials have included patients with mHNPC.

The FinnProstate Study VII randomized 852 locally advanced (50%) or metastatic (50%) PCa to receive ADT for 24 weeks (18). Five hundred fifty-four patients in whom PSA decreased to less than 10 ng/ml, or by 50% or more if less than 20 ng/ml at baseline, were randomized to intermittent (iADT) or continuous ADT (cADT). There were 248 prostate cancer-related deaths: 117 (43%) in the iADT and 131 (47%) in the cADT arms (p = 0.29). This study also investigated HR-Qol with a validated and self-administered 30-item questionnaire addressing ten domains (18). In metastatic patients, ADT improved the pain, activity limitation, and social functioning domain, with iADT further improving activity limitation, social functioning, and recovery of sexual functioning domains.

The South European Urooncological Group (SEUG) trial included 766 patients with locally advanced (67%) or metastatic PCa (30%) who have received a 3-month induction treatment (9). The 626 patients whose PSA level decreased to <4 ng/ml or 80% below the initial value were randomized between iADT and cADT. There was no difference in survival, with an HR of 0.99 (95% CI 0.80-1.23) and 170 deaths in the iADT arm and 169 deaths in cADT arm. Side effects were more pronounced in the continuous arm, and men treated with intermittent therapy reported better sexual function (p < 0.01). The median time off treatment for the patients on iADT was 52 (95% CI 39.4-65.7) weeks.

The intergroup trial SWOG-9346 (EORTC 30985) enrolled 3040 patients with newly diagnosed mHNPC to receive ADT for seven months (8). Of these, 1535 patients achieved a PSA decrease to ≤ 4 ng/mL and were randomized to iADT or cADT. The median follow-up period was 9.8 years. Median survival was 5.8 years in the cADT and 5.1 years in the iADT, resulting in a HR for death with iADT of 1.10 (90% CI 0.99-1.23). The 10% difference in mortality appears to favor the continuous arm, but this did not reach significance. Furthermore, the confidence limits crossed both unity and the pre-determined non-inferiority margin so that non-inferiority was neither proved nor disproved, and the superiority of either arm could not be ruled out. For patients receiving iADT, the median percentage of time on therapy was 47% (IQ range, 23-69). Intermittent treatment was associated with better erectile function and mental health (p<0.001 and p=0.003, respectively) at month three but not after that.

Niraula and Tannock systematically reviewed all the randomized trials comparing iADT to cADT (17). They identified nine studies totaling 5,508 patients treated with either approach. The pooled HR for OS was 1.02 (95% CI 0.94-1.11) for iADT compared with cADT, and the HR for progression-free survival was 0.96 (95% CI 0.76-1.20). Given that neither strategy showed superiority for time-to-event outcomes and iADT was associated with reduced cost, better convenience, and less potential toxicity, they recommended that men with relapsing locally advanced, or metastatic prostate cancer who achieved a good initial response to ADT be treated intermittently rather than continuously. Becker et al. recently performed a meta-analysis of 12 randomized clinical trials using the PRISMA (Preferred Reporting Items for Systematic Reviews and Meta-analyses) guidelines (19). There was no statistically significant difference in prostate cancer specific mortality between intermittent androgen deprivation therapy and continuous androgen deprivation therapy (RR=1.10 [0.85-1.42]) in this mixed population, comprising patients with failure after local treatment and locally advanced or metastatic prostate cancer. The analysis of non-prostate cancer mortality favored intermittent androgen deprivation therapy over continuous androgen deprivation therapy, but the difference was not statistically significant (RR=0.94 [0.76-1.17]).

The registration trials of MAB were designed using continuous administration of both drugs until progression, which is currently the standard of care. However, the continuous use of both drugs results in increased toxicity. In daily practice, physicians, must regularly assess the risk/benefit balance between continuous use or drug holidays. An urgent medical need is to redefine this risk/benefit balance with modern combinations.

## 4 Why a pragmatic clinical trial to revisit iADT?

Registration trials have certain essential limitations.

First, patients are carefully selected to avoid comorbidities and optimize treatment duration. Hence, patients with cardiovascular disease and cognitive changes are often underrepresented. Real-world data collections have shown a significant increase in hospitalization due to cardiovascular complications in patients with pre-existing cardiovascular comorbidities treated with ARPI (20).

Second, pragmatic clinical trials (PrCTs) differ from classical randomized controlled trials (RCTs) as they answer the critical question of the “*effectiveness*” of a treatment in the real-world population rather than its “*efficacy”* in a pre-specified highly selected patient population. PrCTs involve four key design elements: 1) enrolling a *real*-*world population*, 2) being conducted in a *real*-*world setting*, 3) capturing *relevant outcomes* important to inform optimal healthcare treatment decisions and 4) using an *appropriate comparison arm*, which may not always be a placebo treatment. One of the main advantages of PrCTs over real-world observational studies is the use of randomization (21).

Third, unlike explanatory RCTs, PrCTs seek to enroll a broader patient population, including those with comorbidities, adopt more flexible treatment dispensing instructions, and use existing data sources. This leads to more relevant and generalizable findings. Moreover, PrCTs are more likely to incorporate patient-centered outcomes, enabling assessing patient-centered outcomes that are not extensively collected in traditional RCTs. Patient-reported outcomes such as QoL, treatment adherence, and long-term survivorship are critical considerations for individuals living with cancer. By incorporating these outcomes into pragmatic trial designs, clinicians can gain a comprehensive understanding of the impact of interventions on patients’ lives, enabling shared decision-making and improving patient-centered care.

## 5 The challenges of conducting pragmatic clinical trials

As PrCTs target *real-world* patients deemed eligible for a given treatment by their physicians, minimizing the inclusion and exclusion criteria is necessary. Reducing the bureaucracy associated with modern RCTs is crucial to increase pragmatic trial participation by physicians and patients (22). Attempts should be made to reduce the complexity of the study by limiting the number of study procedures and visits, the length of patient questionnaires, or any other deviation from standard of care, routine clinical practice procedures. The idea is to be as close as possible to a real-world setting. The investigator selection should also represent the heterogeneity in clinical practice involving a mix of academic and non-academic centers from different countries.

RCTs are usually conducted in double-blind conditions, with data collection obtained via a strict protocol that must be closely adhered to, to produce robust data. Blinding allows for better reporting of critical endpoints or patients’ outcomes. This does not mimic real-life practice. Because ensuring high-quality data collection in PrCTs is essential, investigators may rely on unified electronic medical records (EMR) to capture prespecified events of interest. Other advantages of EMR include long-term treatment efficacy and safety monitoring at a lower cost. It can also produce data regarding the cost-effectiveness of a given treatment. The De-Escalate trial will incorporate all these principles.

The most critical implementation challenge, not unique to PrCTs, will be patient acceptance of their randomization choice. Patients experiencing toxicity on treatment may refuse to stay on MAB or become silently non adherent. Anxious patients or physicians may find it challenging to adhere to a treatment pause. To mitigate this risk, novel informed consent strategies have been developed, such as the two-stage informed consent, recently introduced by A. Vickers (23, 24).

In the two-stage consent process, patients are *first* informed that they are invited to join a study and discuss data collection and research procedures, such as questionnaires. They are then informed that they might later be randomly selected to hear about an experimental treatment, and if so, they can decide whether to try it or continue with the standard of care. Once baseline data have been collected, randomization will be performed centrally. Patients who signed the first consent and are not randomized to the experimental arm will stay in the control arm, receiving standard-of-care treatment. In De-Escalate, patients with deep PSA response will first consent to the collection of data. Then, within a month of the randomization, patients randomly selected to the intermittent treatment arm will be informed about the investigational treatment (here iMAB) and are asked to sign a second informed consent. If they refuse, they will stay on the control arm (cMAB) and the only addition to their clinical routine will be to complete HR-QoL questionnaires at regular intervals. PSA, testosterone, further anticancer treatment, and grade 3 to 5 toxicity will be collected from routine follow-up. Following the intent-to-treat principle, patients are analyzed in the experimental arm irrespective of their decision at this second stage. Vickers et al. have reported that conducting consent in two stages reduces the decisional anxiety, confusion, and information overload commonly associated with informed consent (23, 24). Although this raised some concerns during an advisory ethical committee review, with further explanations and endorsement from patients’ organization Europa Uomo, it was found acceptable.

Finally, a last major hurdle we observed while developing the study design was its recognition as a low interventional clinical trial. This is defined by EU clinical trial regulation (CTR) when 1) the interventional medicine product (IMP) is authorized and considered as SOC, 2) the use of the IMP is evidence-based and supported by robust scientific evidence on its safety and efficacy and 3) the additional diagnostic or monitoring procedures do not pose more than minimal additional risk or burden to the safety of the subjects compared to normal clinical practice. De-Escalate trial will not mandate the use of any specific ADT or ARPI drug. But instead, all medications currently used as standard of care under their marketing authorization, depending on the participating countries, will be available as treatment options, addressing the first two point of the definition of a low interventional clinical trial. Since there are no extra tests or imaging required, and monitoring of PSA and testosterone level is routinely done, there is no extra burden for the patients. We faced however some issues regarding the intermittent use of both ADT and ARPI or “treatment holiday”, as in registration trials, regulators requested that patients would be treated continuously for methodological reasons.

## 6 Assessing the “pragmatism” of a trial

Not every trial can be pragmatic. The PRECIS-2 criteria “PRagmatic-Explanatory Continuum Indicator Summary v2” have been developed to help investigators develop more pragmatic trials (25). It evaluates a trial’s design via several key dimensions: recruitment of participants (no exclusion criteria based on upper age limit, comorbidities, …) and investigators, the setting of the trial (multiple centers, academic and non-academic), the delivery and adherence to the protocol (flexibility in monitoring of compliance), follow-up (minimum number of FU visits), endpoints (relevant for patients such as OS or QoL) and analysis (all data are included in the analysis)?. Each dimension is scored between 0 to 5, with 5 being the most pragmatic.

**Figure 2:**
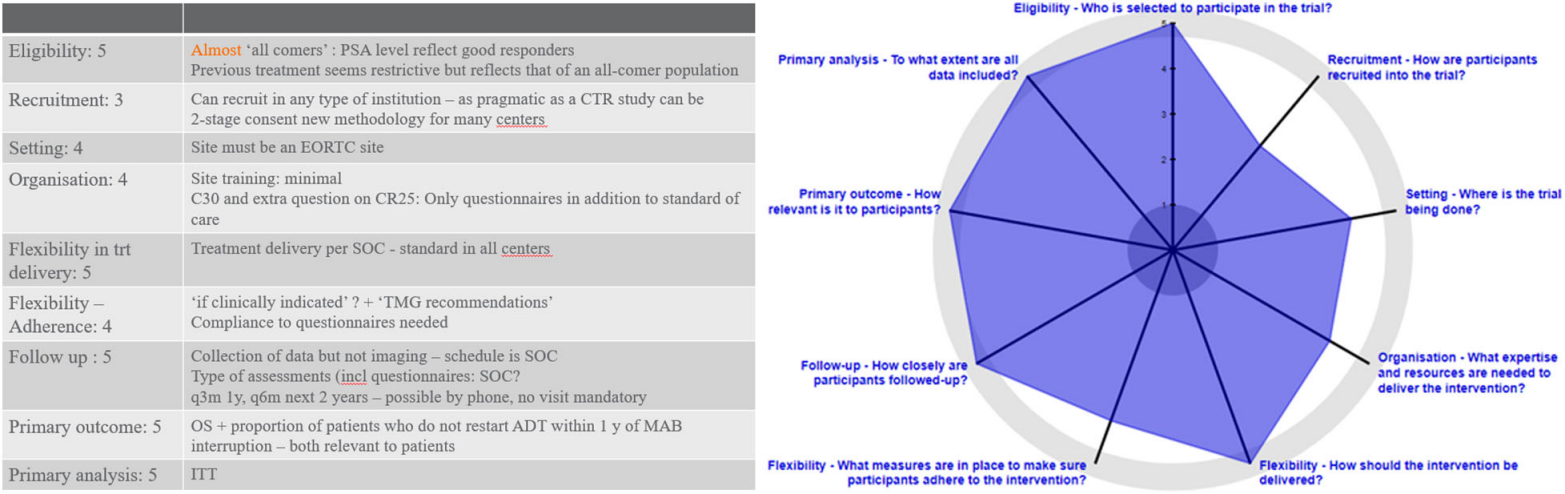
PRECIS-2 evaluation of EORTC-2238.

We tested the PRECIS-2 tool with EORTC 2238 De-Escalate (**Figure 2**). It demonstrates the trials pragmatic approach, as it scores 5 points in eligibility, flexibility in treatment delivery, follow-up, primary outcome and primary analysis. It scores 4 in setting of the trial, adherence and organization. The only dimension to score 3 points is recruitment of patients. **Figure 2** summarizes each dimension.

## 7 Conclusion

Revisiting the concept of intermittent treatment in the era of combined treatment for metastatic hormone-naïve prostate cancer is a critical challenge. Routinely combining AR pathway inhibitors with androgen deprivation therapy does indeed significantly prolong survival but at the price of more toxicity and resource utilization. Patients with a profound PSA response deserve that we investigate a better balance between treatment duration, toxicity, and survival. Such trials require many patients from many countries, hence large resources.

Pragmatic trials have emerged as a valuable tool for generating evidence that could guide clinical practice and health policy decisions. These trials can improve treatment outcomes, enhance patient care, and optimize resource allocation by including diverse patient populations, evaluating patient-centered outcomes, and assessing implementation strategies. However, challenges include methodology issues, data collection quality, and balancing protocol flexibility with scientific rigor.

The EORTC 2238 De-Escalate trial will revisit the concept of intermittent treatment in patients with a deep PSA response after 6 to 12 months of induction treatment, seeking to spare them from chronic toxicities while maintaining their overall survival and improving their quality of life.

## Data availability statement

The original contributions presented in the study are included in the article/supplementary material. Further inquiries can be directed to the corresponding author.

## Author contributions

GG: Writing – review & editing, Writing – original draft. FT: Writing – review & editing, Writing – original draft. SL: Writing – review & editing, Writing – original draft. BF: Writing – review & editing, Writing – original draft. AP: Writing – review & editing, Writing – original draft. EG: Writing – review & editing, Writing – original draft. RM: Writing – review & editing, Writing – original draft. AA: Writing – review & editing, Writing – original draft. SG: Writing – review & editing, Writing – original draft. BT: Writing – review & editing, Writing – original draft.

## References

[1] Fizazi K Tran N Fein L Matsubara N Rodriguez-Antolin A Alekseev BY. Abiraterone plus prednisone in metastatic, castration-sensitive prostate cancer. N Engl J Med. (2017) 377:352–60. doi: 10.1056/NEJMoa1704174 28578607

[2] James ND de Bono JS Spears MR Clarke NW Mason MD Dearnaley DP. Abiraterone for prostate cancer not previously treated with hormone therapy. N Engl J Med. (2017) 377:338–51. doi: 10.1056/NEJMoa1702900 PMC5533216 28578639

[3] Fizazi K Foulon S Carles J Roubaud G McDermott R Fléchon A. Abiraterone plus prednisone added to androgen deprivation therapy and docetaxel in *de novo* metastatic castration-sensitive prostate cancer (PEACE-1): a multicentre, open-label, randomised, phase 3 study with a 2× 2 factorial design. Lancet. (2022) 399:1695–707. doi: 10.1016/S0140-6736(22)00367-1 35405085

[4] Chi KN Chowdhury S Bjartell A Chung BH Pereira de Santana Gomes AJ Given R. Apalutamide in patients with metastatic castration-sensitive prostate cancer: final survival analysis of the randomized, double-blind, phase III TITAN study. J Clin Oncol. (2021) 39:2294–303. doi: 10.1200/JCO.20.03488 33914595

[5] Davis ID Martin AJ Stockler MR Begbie S Chi KN Chowdhury S. Enzalutamide with standard first-line therapy in metastatic prostate cancer. N Engl J Med. (2019) 381:121–31. doi: 10.1056/NEJMoa1903835 31157964

[6] Armstrong AJ Szmulewitz RZ Petrylak DP Holzbeierlein J Villers A Azad A. ARCHES: a randomized, phase III study of androgen deprivation therapy with enzalutamide or placebo in men with metastatic hormone-sensitive prostate cancer. J Clin Oncol. (2019) 37:2974–86. doi: 10.1200/JCO.19.00799 PMC6839905 31329516

[7] Smith MR Hussain M Saad F Fizazi K Sternberg CN Crawford ED. Darolutamide and survival in metastatic, hormone-sensitive prostate cancer. N Engl J Med. (2022) 386:1132–42. doi: 10.1056/NEJMoa2119115 PMC9844551 35179323

[8] Hussain M Tangen CM Berry DL Higano CS Crawford ED Liu G. Intermittent versus continuous androgen deprivation in prostate cancer. N Engl J Med. (2013) 368:1314–25. doi: 10.1056/NEJMoa1212299 PMC3682658 23550669

[9] Calais da Silva FE Bono AV Whelan P Brausi M Marques Queimadelos A Martin JA. Intermittent androgen deprivation for locally advanced and metastatic prostate cancer: results from a randomised phase 3 study of the South European Uroncological Group. Eur Urol. (2009) 55:1269–77. doi: 10.1016/j.eururo.2009.02.016 19249153

[10] Crook JM O'Callaghan CJ Duncan G Dearnaley DP Higano CS Horwitz EM. Intermittent androgen suppression for rising PSA level after radiotherapy. N Engl J Med. (2012) 367:895–903. doi: 10.1056/NEJMoa1201546 22931259 PMC3521033

[11] Hussain M Goldman B Tangen C Higano CS Petrylak DP Wilding G. Prostate-specific antigen progression predicts overall survival in patients with metastatic prostate cancer: data from Southwest Oncology Group Trials 9346 (Intergroup Study 0162) and 9916. J Clin Oncol. (2009) 27:2450–6. doi: 10.1200/JCO.2008.19.9810 PMC2684851 19380444

[12] Saad F Hussain M Tombal B Fizazi K Sternberg C Crawford D. Association of prostate-specific antigen (PSA) response and overall survival (OS) in patients with metastatic hormone-sensitive prostate cancer (mHSPC) from the phase 3 ARASENS trial. JCO. (2022) 40:5078–8. doi: 10.1200/JCO.2022.40.16_suppl.5078

[13] Gillessen S Bossi A Davis ID de Bono J Fizazi K James ND. *M*anagement of patients with advanced prostate cancer—metastatic and/or castration-resistant prostate cancer: report of the Advanced Prostate Cancer Consensus Conference (APCCC) 2022. Eur J Cancer. (2023) 185:178–215. doi: 10.1016/j.ejca.2023.02.018 37003085

[14] Rizzo A Merler S Sorgentoni G Oderda M Mollica V Gadaleta-Caldarola G. Risk of cardiovascular toxicities and hypertension in nonmetastatic castration-resistant prostate cancer patients treated with novel hormonal agents: a systematic review and meta-analysis. Expert Opin Drug Metab Toxicol. (2021) 17:1237–43. doi: 10.1080/17425255.2021 34407702

[15] Myint ZW Momo HD Otto DE Yan D Wang P Kolesar JM. Evaluation of fall and fracture risk among men with prostate cancer treated with androgen receptor inhibitors: a systematic review and meta-analysis. JAMA Netw Open. (2020) 3:e2025826. doi: 10.1001/jamanetworkopen.2020.25826 33201234 PMC7672516

[16] Nowakowska MK Ortega RM Wehner MR Nead KT. Association of second-generation antiandrogens with cognitive and functional toxic effects in randomized clinical trials: A systematic review and meta-analysis. JAMA Oncol. (2023) 9:930–7. doi: 10.1001/jamaoncol.2023.0998 PMC10214180 37227736

[17] Niraula S Le LW Tannock IF. Treatment of prostate cancer with intermittent versus continuous androgen deprivation: a systematic review of randomized trials. J Clin Oncol. (2013) 31:2029–36. doi: 10.1200/JCO.2012.46.5492 23630216

[18] Salonen AJ Taari K Ala-Opas M Viitanen J Lundstedt S Tammela TL. Advanced prostate cancer treated with intermittent or continuous androgen deprivation in the randomised FinnProstate Study VII: quality of life and adverse effects. Eur Urol. (2013) 63:111–20. doi: 10.1016/j.eururo.2012.07.040 22857983

[19] Becker B Stroever S Reddy A de Riese WTW. Comparison of intermittent and continuous androgen deprivation therapy in prostate cancer patients: an up-to-date meta-analysis for urologists and medical providers. Urol Pract. (2023) 10:424–34. doi: 10.1097/UPJ.0000000000000424 37505912

[20] Lu-Yao G Nikita N Keith SW Nightingale G Gandhi K Hegarty SE. Mortality and hospitalization risk following oral androgen signaling inhibitors among men with advanced prostate cancer by pre-existing cardiovascular comorbidities. Eur Urol. (2020) 77:158–66. doi: 10.1016/j.eururo.2019.07.031 PMC6980462 31420248

[21] Gamerman V Cai T Elsäßer A. Pragmatic randomized clinical trials: best practices and statistical guidance. Health Serv Outcomes Res Method. (2019) 19:23–35. doi: 10.1007/s10742-018-0192-5

[22] Gribben J Macintyre E Sonneveld P Doorduijn J Gisselbrecht C Jäger U. Reducing bureaucracy in clinical research: a call for action. Hemasphere. (2020) 4:e352. doi: 10.1097/HS9.0000000000000352 32309789 PMC7162089

[23] Vickers AJ Vertosick EA Carlsson SV Ehdaie B Kim SYH. Patient accrual and understanding of informed consent in a two-stage consent design. Clin Trials. (2021) 18:377–82. doi: 10.1177/1740774520988500 PMC8363154 33530713

[24] Vickers AJ Young-Afat DA Ehdaie B Kim SY. Just-in-time consent: the ethical case for an alternative to traditional informed consent in randomized trials comparing an experimental intervention with usual care. Clin Trials. (2018) 15:3–8. doi: 10.1177/1740774517746610 PMC5799028 29224379

[25] Loudon K Treweek S Sullivan F Donnan P Thorpe KE Zwarenstein M. The PRECIS-2 tool: designing trials that are fit for purpose. BMJ. (2015) 350:h2147. doi: 10.1136/bmj.h2147 25956159

